# Grafting Poly(Methyl Methacrylate) (PMMA) from Cork via Atom Transfer Radical Polymerization (ATRP) towards Higher Quality of Three-Dimensional (3D) Printed PMMA/Cork-*g*-PMMA Materials

**DOI:** 10.3390/polym12091867

**Published:** 2020-08-19

**Authors:** Paula S. S. Lacerda, Nuno Gama, Carmen S. R. Freire, Armando J. D. Silvestre, Ana Barros-Timmons

**Affiliations:** CICECO—Aveiro Institute of Materials and Department of Chemistry, University of Aveiro—Campus Santiago, 3810-193 Aveiro, Portugal; placerda@ua.pt (P.S.S.L.); nuno.gama@ua.pt (N.G.); cfreire@ua.pt (C.S.R.F.); armsil@ua.pt (A.J.D.S.)

**Keywords:** cork, poly(methyl methacrylate), atom transfer radical polymerization (ATRP), blends, interface, mechanical properties, 3D-printing

## Abstract

Cork is a unique material and its by-products are attracting an ever-growing interest for preparing new materials in an attempt to extend the outstanding properties of cork toward innovative and high value applications. Yet, the miscibility of cork particles with thermoplastic matrices is not easy due to its low density and surface properties. Here, cork is functionalized with poly(methyl methacrylate) (PMMA) via atom transfer radical polymerization (ATRP) to yield cork grafted with PMMA chains particles (cork-*g*-PMMA). Both the ATRP macroinitiator and the cork-*g*-PMMA obtained are fully characterized by Fourier-transform infrared spectroscopy (FT-IR), ^13^C cross-polarized magic-angle spinning solid-state nuclear magnetic resonance (^13^C CP/MAS solid state NMR), scanning electron microscopy with energy dispersive X-ray spectroscopy (SEM-EDX), X-ray diffraction (XRD) and thermogravimetric analyses (TGA). The functionalized cork particles are then blended with commercial PMMA to afford cork-*g*-PMMA/PMMA. To compare, cork also is mixed with PMMA and the ensuing cork/PMMA sample and its morphology, thermal, and mechanical properties are compared with those of cork-*g*-PMMA/PMMA and commercial PMMA. The cork surface modification via ATRP of the methyl methacrylate (MMA) yields better dispersion in the matrix. Consequently, a blend with enhanced mechanical performance, higher thermal stability, and a higher melt flow index (MFI) is obtained when compared to the blend prepared using unmodified particles. The similarity of the MFI of cork-*g*-PMMA/PMMA to that of PMMA suggests good printability. Indeed, a three-dimensional (3D) printed specimen is obtained confirming that grafting using ATRP is a promising route for the preparation of high quality 3D printed products.

## 1. Introduction

Cork, the outer bark of *Quercus suber* L., is a plant tissue composed of suberin (30–60%), lignin (19–22%), polysaccharides (12–20%) and extractives (9–20%) [1] and has unique properties such as a very low density, hydrophobic character, fire resistance, low thermal and acoustic conductivity and elastic behavior [1,2]. The cork industry produces annually approximately 200,000 tons of cork worldwide [3], mainly to produce cork stoppers, as well as materials for thermal and acoustic insulation [4,5,6,7,8,9]. During processing, large amounts of cork (20–30%) are converted into a low granulometric powder, the so-called cork powder that so far has almost no added value application apart from energy conversion. Therefore, the valorization of this industrial residue as a source of new added-value chemicals and polymers, and for the development of new added-value materials via the preparation of composites, has been receiving increasing interest [9,10,11,12,13].

Cork based polymer composites have been developed by different groups using both synthetic as well as natural matrices [14,15,16,17,18,19], bringing together the need to develop greener materials while exploring the unique properties of cork mentioned above. Whilst good dispersion and good cork-matrix adhesion have been reported when biobased polymers were used, as in the case of synthetic hydrophobic polymer matrices, namely polyolefins such as polypropylene or polyethylene, the use of compatibilizers and/or surface treatment was required to obtain good dispersion and mechanical performance [15,20,21,22]. An alternative route to modify the surface properties of fillers to achieve good dispersion in polymer matrices, as well as good adhesion, involves the grafting of polymer chains to or from the surface of fillers, which has been proposed by various authors to improve the properties of the ensuing composite materials [23]. Additionally, this type of surface modification may help increase the thickness of the effective interphase zone (i.e., the interface zone behaving like the filler) and, therefore, modulate the effective volume fraction of the fillers [24].

While a variety of strategies have been used to graft polymers onto fillers, the use of reversible-deactivation radical polymerization (RDRP) mechanisms allows for growing polymer chains with controlled molecular weights, composition, and functionality, among other features. During the last 25 years, atom transfer radical polymerization (ATRP) is one of the most used polymerization mechanisms and has been recently reviewed [25,26]. It allows for the use of a diversity of functional monomers, of reaction conditions, and has the ability to produce polymers with high chain-end functionalities, making this strategy very convenient for the preparation of novel materials with tailor-made functionalities by judicious choice of the polymeric blocks. Furthermore, surface-initiated ATRP has been proven to be a versatile method for grafting polymers from surfaces containing hydroxyl groups, which can be easily functionalized to α-bromoesters and are excellent ATRP initiators [27,28,29].

Normally, the production of composite-based products involves conventional processing methods such as injection molding or extrusion. However, new processing methods are emerging, such as additive manufacturing (i.e., three-dimensional (3D) printing), which can be used to produce biomedical, aerospace, automotive, electronic, architectural, fashion, or domestic products [30]. Moreover, using this technology it is possible to produce products with new forms, shapes, and dimensions, which are impossible to obtain using conventional methods [31]. The use of this technology in industry also has the advantage of not requiring the production of prototypes. However, in the case of composite materials, the wettability of the fillers by the matrix is critical to ensure adequate viscosity during printing as well as layer-to-layer adhesion [32].

During this study, cork residues are modified with poly(methyl methacrylate) chains grown via ATRP to enhance their compatibility with the polymer matrix. To assess the relevance of the surface modification strategy, blends of unmodified and modified cork particles with commercially available poly(methyl methacrylate) (PMMA) are prepared. These materials are fully characterized by a series of techniques which confirm the successful modification of cork particles, and the enhanced thermal and mechanical properties of the blends containing cork-*g*-PMMA. Finally, the potential of the prepared materials is demonstrated in 3D printing assays.

## 2. Materials and Methods

### 2.1. Materials

All chemicals were of analytical grade and were used as received unless otherwise stated, except for the methyl methacrylate (MMA) monomer which was passed through a column of neutral aluminium oxide (CARLO ERBA Reagents, Chaussée du Vexin, Val-de-Reuil, France, particle size 63–200 μm) prior to use to remove the inhibitor. Dimethylformamide (DMF) was dried using activated molecular sieves. Poly(methyl methacrylate) (PMMA) with a M_w_ of 350,000 and a *T_g_* of 122.0 °C was supplied by Sigma–Aldrich. Methyl acetyl ricinoleate (Flexricin™ P-4), with a specific gravity of 0.94 g.cm^−3^ and a viscosity of 20 cP (at 25 °C), was used as plasticizer and supplied by Vertellus.

### 2.2. Preparation of Cork Material

Cork, supplied by Corticeira Amorim, S.A (Portugal), was finely ground using an agate vibratory disk mill and then passed through a 125 μm particle sieve. This material was used as a model of industrial cork powder. Prior to modification, lipophilic and polar cork extractives were removed as described elsewhere [33,34]; briefly, the cork powder was extracted in a Soxhlet with dichloromethane for 8 h; the solid residue was then transferred to a flat-bottomed flask and suspended in a methanol:water (MeOH:H_2_O) mixture, 50/50 (*v*/*v*), for 24 h at room temperature under constant stirring. The suspension was then filtered, dried at 40 °C in a vacuum oven and kept in a desiccator (please see results and discussion for the rationale of extractive removal).

### 2.3. Preparation of Cork Macroinitiator (C-BiB)

Both esterification and grafting (Section 2.4) methodologies were adapted from procedures described elsewhere [27]. Cork (1.0 g) was introduced into a round-bottomed flask equipped with a rubber septum, containing 4-(dimethylamino)-pyridine (DMAP) (4 mmol). Anhydrous *N*,*N*-dimethylformamide (DMF) (33 mL) then was introduced and the resulting suspension was degassed by bubbling N_2_ for 30 min under constant stirring. The reaction flask was put into an ice bath and 2-bromoisobutyryl bromide (B*i*BB) (2 mmol) was added dropwise. The resulting suspension was then left to thaw up to room temperature and kept under stirring for two hours. The ensuing cork macroinitiator (C-B*i*B) was filtrated and dried under vacuum.

### 2.4. Grafting of Methyl Methacrylate from Initiator-Functionalized Cork

A Cu-PMDETA stock solution was prepared in a glass vial by vigorously dispersing copper(I) bromide (Cu(I)Br, 0.186 mmol) in degassed water (1.2 mL) followed by the addition of *N*,*N*,*N*′,*N*″,*N*″-pentamethyldiethylenetriamine (PMDETA, 0.186 mmol). Degassed DMF (4.9 mL) was added to C-B*i*B (0.5 g) in a round-bottomed flask equipped with a rubber septum. To this suspension, the freshly prepared catalytic solution was added, and the resulting mixture was purged with N_2_ for 30 min. Finally, previously degassed MMA (10 mL; 0.19 mol·g^−1^ of cork) was injected into the reaction mixture, which was then immersed in an oil bath at 50 °C for 4.5 h under constant stirring. The polymerization was stopped by exposure to air and the resulting material was washed by filtration with DMF and dichloromethane.

### 2.5. Weight-Gain Calculation

The cork polymerization weight gain (*G*, wt%) was calculated using Equation (1).

(1) $$G=\left(\left(W_{2}-W_{1}\right)/W_{1}\right)\times 100$$

where $W_{1}$ is the cork macroiniciator weight and $W_{2}$ the weight after polymerization.

### 2.6. Preparation of Blends

The poly(methyl methacrylate) powder was mixed with 75 parts per hundred of resin (phr) of methyl ricinoleate [35,36] in a Felfil Evo Colours extruder using 4 rpm at 175 °C to produce the 3D printable filaments. The cork-based filaments were produced in the same way, adding cork powder or C-*g*-PMMA (5% *wt*/*wt* which, according to a previous report [9], is enough to improve the properties of the ensuing materials without compromising their processability). The resulting filaments were used to 3D print specimens (length of 50 mm, width of 10 mm and thickness of 5 mm) in an Anycubic Chiron 3D printer (Shenzhen, China) using the 3D print parameters presented in Table 1. TopSolid Missler Software (Evry, France) was used to design the sketch of specimens and Ultimaker Cura (UK) was used to prepare the 3D printing model.

### 2.7. Characterization Methods

The ^13^C solid-state cross-polarized magic-angle spinning nuclear magnetic resonance (^13^C CP/MAS NMR) spectra were recorded at 9.4 T using a frequency of 400 MHz on a Bruker Avance 400 spectrometer using a 4 mm double-bearing probe, 9 KHz spinning rate, and MAS with proton 90° pulses of 40 μs [37].

The attenuated total reflectance Fourier-transform infrared spectroscopy (ATR-FTIR) spectra were acquired using a Perkin Elmer FTIR System Spectrum BX Spectrometer (MA, USA) equipped with a single horizontal Golden Gate ATR cell. Thirty-two scans were acquired in the 4000−500 cm^−1^ range with a resolution of 1 cm^−1^. All spectra were normalized for the highest band in the acquired range.

A Setsys Evolution 1750 (Setaram, France) was used to carry out thermogravimetric analyses (TGA) from room temperature up to 800 °C, at a heating rate of 10 °C·min^−1^, under nitrogen flow (20 mL·min^−1^).

Scanning electron microscopy (SEM) images were obtained using a Hitachi SU-70 SEM (Tokyo, Japan), and energy dispersive X-ray spectroscopy (EDX) analysis was performed using an EDX Bruker Quantax 400 (Billerica, MA, USA). Specimens were coated with carbon prior to analysis.

X-ray diffraction (XRD) patterns were collected using a Philips X’pert MPD diffractometer (Eindhoven, Netherlands) using Cu Kα radiation. The scattered radiation was detected in the angular range from 4° to 60° (2*θ*).

The MFI was determined following the ISO 1133-1:2011 standard [38] using a Model 6 Advanced melt flow system of Ray–Ran (Warwickshire, UK). After packing the material inside the barrel and heating it to 190 °C a piston (of 5 kg) was introduced into the barrel, which caused the extrusion of the molten polymer. The samples of the melted polymer were weighed, and MFI expressed in grams of polymer per 10 min of duration of the test.

Stress–strain tests of filaments were performed in a SHIMADZU AGS-X (Kyoto, Japan) using a load cell of 1 kN and a deformation rate of 5.0 mm·min^−1^. Three specimens were tested for each sample.

Dynamic mechanical analyses (DMA) were carried out using a Tritec 2000 equipment (Scotland, UK). The filaments and 3D printed specimens were analyzed in tension mode from 0 °C up to 125 °C at a constant heating rate of 2 °C·min^−1^ and at a frequency of 1 Hz.

## 3. Results

**Scheme 1:**
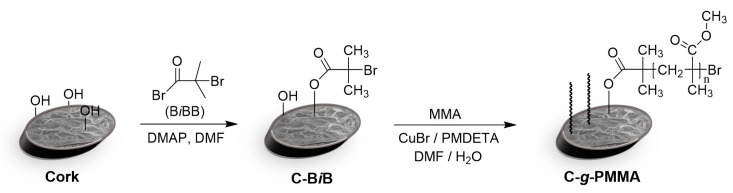
Grafting of cork with PMMA; C-B*i*B: cork functionalized with 2-bromoisobutyryl (B*i*B) moieties, C-*g*-PMMA: cork grafted with PMMA.

Cork hybrids were obtained by a “grafting from” method in two steps (Scheme 1).

First, cork was functionalized with 2-bromoisobutyryl (B*i*B) moieties acting as ATRP initiator groups. During the second step, methyl methacrylate (MMA) was grafted from the cork macroinitiator. These procedures were performed in heterogeneous media using extractive free cork powder. During a preliminary study (data not shown), polymerization was promoted in ground cork (not extracted) and grafting was achieved. However, during these experimental procedures, the liquid fractions always presented a brownish color indicating the release of components from cork. Considering the complex chemical composition of cork and the possibility of the release of anchoring points (if bonded to extractives), it was decided to remove cork from its extractables before use.

### 3.1. Macroinitiator Synthesis

**Figure 1:**
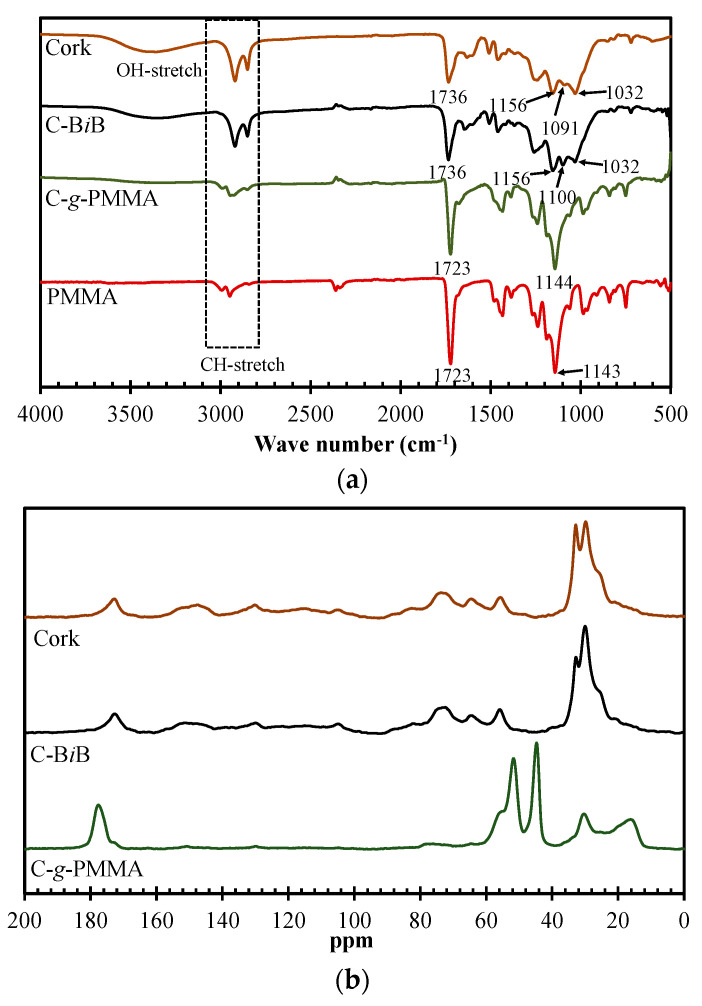
ATR-FTIR (**a**) and ^13^C CP/MAS solid state NMR (**b**) of Cork, C-B*i*B, C-*g*-PMMA and ATR-FTIR of homopolymer PMMA (produced via ATRP).

Cork macroinitiator (C-B*i*B) was synthesized through acylation of hydroxyl groups containing components (mainly polysaccharides and lignin) with bromoisobutyryl bromide. The comparison of the ATR-FTIR spectra (Figure 1a) of cork, before and after acylation, confirmed the functionalization with ATRP initiators by the small decrease in the intensity of the broad band at about 3350 cm^−1^ assigned to the hydroxyl groups stretching, indicating the partial substitution of these groups. The band at 1736 cm^−1^ is mainly attributed to an ester carbonyl group stretching band from the cork’s suberin groups, yet it exhibits a slight increase associated with the contribution of the same type of vibrations from the 2-bromoisobutyryl groups [37].

The C-B*i*B also was characterized by ^13^C CP/MAS solid state NMR. The obtained spectrum (see Figure 1b) shows a profile similar to the one of the parent extractives’ free cork material with two major resonances in the aliphatic area at δ 29.8 and 32.9 ppm, attributed to methylenic carbons of suberin’s typical long aliphatic carbon chains [37,39]. The spectra also show the resonance at δ 55.7 ppm attributed to methoxyl groups of lignin, suberin and hemicelluloses. Seen in the range of δ 64.6–105.0 ppm, the resonances are mainly from cellulose and hemicellulose carbons with overlaps from lignin and suberin [37,39], while from δ 109.3 to 152.2 ppm, the resonances are due to lignin aromatic carbons. The esterified nature of cork is indicated by the resonance at δ 172.8 ppm assigned to carbonyl carbons of ester groups from suberin and lignin cork components [37,39]. Similar to that observed for the macroinitiator ATR-FTIR spectrum, no significant change is observed in this area of the spectrum, pointing to a low degree of acylation by bromoisobutyryl bromide.

**Figure 2:**
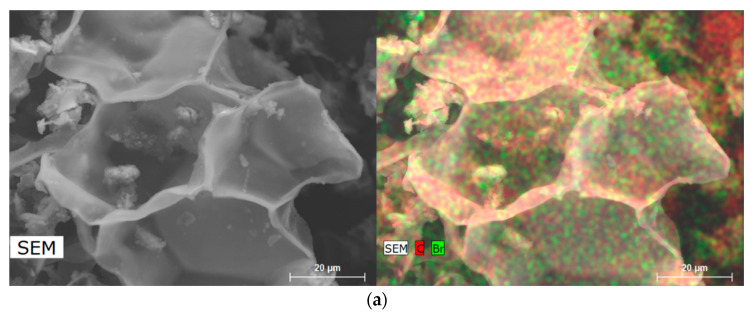

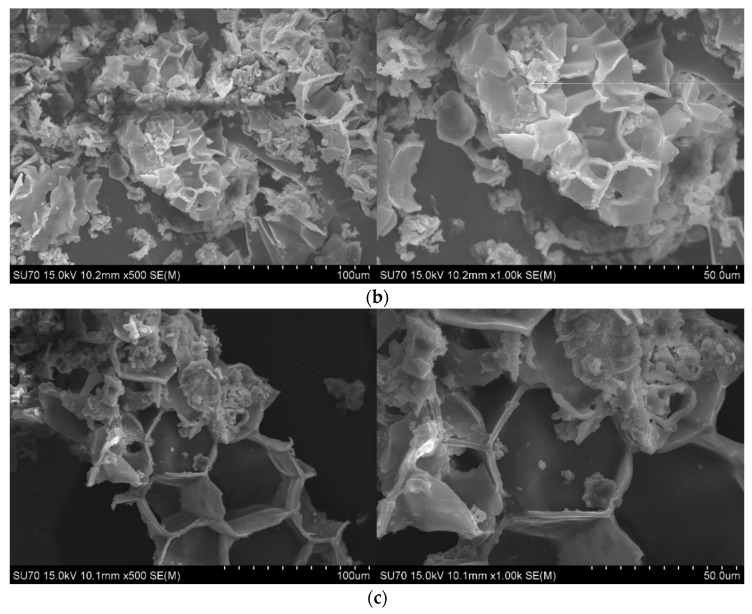
SEM image and EDX bromine mapping of C-B*i*B (**a**) and SEM image of Cork (**b**) and C-*g*-PMMA (**c**).

Further evidence of initiator group grafting also is provided by SEM energy dispersive X-ray analysis with the presence of bromine (Br) peak, and bromine mapping (Figure 2a) showing a uniform distribution of bromine (as green dots) throughout the material.

Finally, it is worth mentioning, based on SEM analysis, cork pristine morphology, particularly its alveolar structure, is largely preserved in C-B*i*B and C-*g*-PMMA (despite the mass gain discussed below), thus allowing us to infer that most relevant cork properties will be preserved.

### 3.2. Grafting of MMA from Cork Macroinitiator

Using the C-B*i*B macroinitiator prepared above, ATRP was carried out in a water:DMF (80:20) mixture at 50 °C under nitrogen using Cu(I)Br/PMDETA as a catalyst system. Upon extensive washing, grafting of MMA from cork led to an increase in its dry weight, affording C-*g*-PMMA with a 205% weight gain. The ATR-FTIR spectrum of the ensuing material is presented in Figure 1a. To compare, the spectrum of PMMA homopolymer synthesized under similar reaction conditions also is presented. Comparison of the ATR-FTIR spectra of cork, before and after polymerization, confirms PMMA grafting. The C-*g*-PMMA spectrum shows strong peaks due to the carbonyl stretch of the ester at 1723 cm^−1^ and at 1144 cm^−1^ due to the ester C=O and C–O stretching respectively, confirming the presence of PMMA [37,39] and, also, the disappearance of the suberin carbonyl ester at 1736 cm^−1^ [37,39] of the broad band at about 3350 cm^−1^ assigned to the O–H stretching [37,39] and a substantial reduction of the band at about 2850 cm^−1^ and 2919 cm^−1^ assigned to the C–H groups [37,39]. Moreover, the C-*g*-PMMA spectrum profile is very similar to the one of the PMMA homopolymer, indicating an extensive coverage of the cork material by PMMA [27].

Further evidence of the cork grafting is provided by the ^13^C CP/MAS solid state NMR of the ensuing material, shown in Figure 1b. The C-*g*-PMMA spectrum shows typical PMMA resonances at δ 16.2 ppm due to α-CH_3_ [27], 44.7 ppm due to the quaternary carbon [27], 55.7 ppm due to OCH_3_ together with a shoulder at 55.0 ppm due to CH_2_ [27], and δ 177.6 ppm due to the resonance of the carbonyl carbon [27]. This spectrum also shows at δ 30.3 ppm [37] the resonance due to cork’s suberin chain CH_2_—the main resonance on ungrafted cork, (Figure 1b) and the resonance of cork’s carbonyl carbons at δ 177.1 ppm as a shoulder [37]. The higher intensity of PMMA resonances over the cork-originated ones on the ^13^C NMR spectrum of the grafted material is in line with the previously discussed FTIR data and weight gain observed upon cork polymerization.

The morphology of the unmodified and grafted cork was assessed by SEM and the images obtained are presented in Figure 2b,c. The starting material shows the typical cellular morphology of cork along with bulk random structures that might have resulted from the cork grinding procedure. Actually, and despite the substantial mass gain observed for C-*g*-PMMA, no radical changes in the surface morphology of cork before and after modification were registered, since its morphology was preserved.

**Figure 3:**
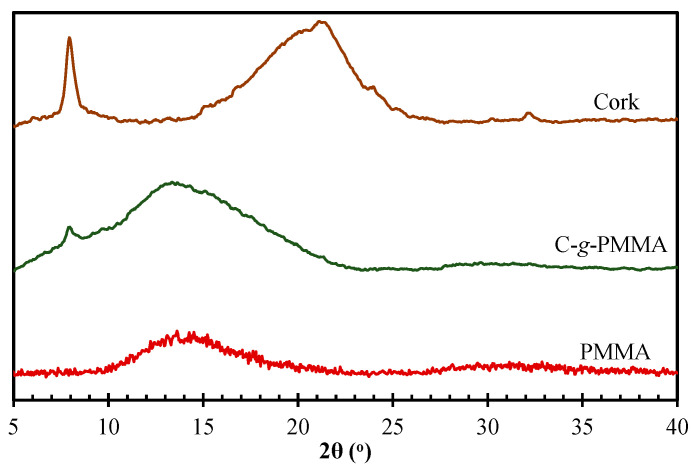
X-ray diffractograms of Cork, C-*g*-PMMA and PMMA homopolymer (produced via ATRP).

The XRD diffractograms of ungrafted and grafted cork are exhibited in Figure 3, as well as the one of PMMA homopolymer prepared via ATRP (presented for comparison). The X-ray diffraction pattern displayed by cork is typical of amorphous materials, with a broad amorphous halo (broad band centered at approximately 2*θ* ≈ 22°) [37]. The peak at 2*θ* = 8.0° could have originated from contamination during the grinding procedure with an agate vibratory disk mill.

The C-*g*-PMMA exhibited a similar diffraction pattern, but with the diffraction maximum at approximately 2*θ* ≈ 14° due to the grafted PMMA of this new material [37]. The same maximum of 2*θ* is observed for the PMMA homopolymer (see Figure 3).

**Figure 4:**
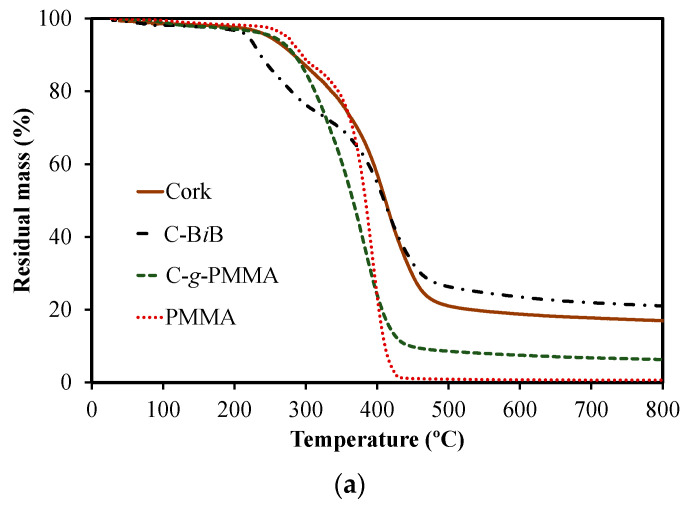

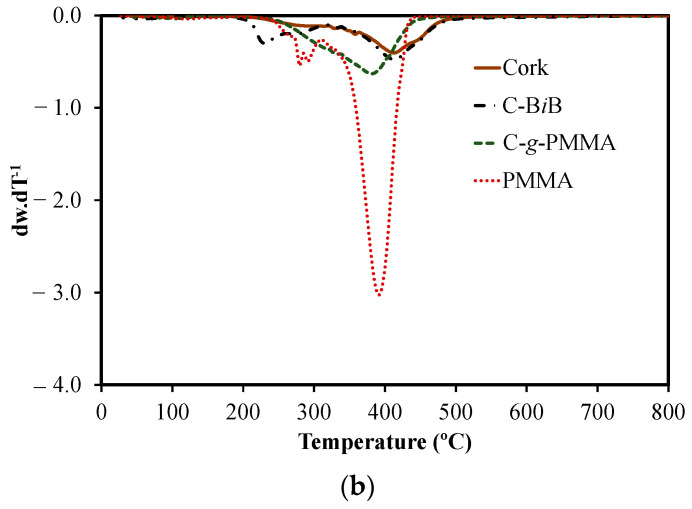
Thermal analyses: thermogram (**a**,**b**) first derivative of weight loss as a function of temperature of Cork, C-B*i*B, C-*g*-PMMA and PMMA homopolymer (produced via ATRP).

The thermal stability and degradation profile of the final grafted material, corresponding intermediates, and starting material were assessed by TGA as summarized in Table 2 and Figure 4. The TGA and corresponding derivative of weight loss as a function of temperature (DTG) profiles of the PMMA homopolymer are also presented for comparison purposes.

The extractive-free cork used as the starting material for this study is thermally stable up to 291 °C (*T*_10%, deg_) and exhibits a gradual multi-step weight loss profile typical of complex biomass-based materials; the maximum decomposition rate was reached at 412 °C. C-B*i*B showed a reduction of the thermal stability to 243 °C (*T*_10%, deg_) and two main degradation steps at 230 °C and 413 °C (*T*_max, deg_). These thermal degradation effects are attributed to the introduction of B*i*B groups and has been reported for this type of macroinitiator obtained with other lignocellulosic and cellulosic materials [27]. Furthermore, PMMA grafting from the cork macroinitiator imparted thermal stability to the ensuing material as *T*_10%, deg_ increased to 287 °C, a value similar to that of unmodified cork. A distinct degradation profile was detected, however, for C-*g*-PMMA, showing a single mass-loss step process and maximum decomposition rate at 381 °C. This value is lower than that obtained for both cork and C-B*i*B, reflecting the effect of the grafted PMMA on the material. This is confirmed by the fact that the maximum decomposition rate of PMMA homopolymer occurs at 391 °C. The solid residue remaining at 800 °C accounted for 16.9, 21.0 and 6.3 wt% for cork, C-B*i*B and C-*g*-PMMA, respectively. The reduction of the residue remaining at the end of the TGA experiment for C-*g*-PMMA was expected and is in agreement with the weight gain (*G* = 205% value) registered for this material and the fact that the PMMA moiety undergoes almost complete decomposition, as indicated by the thermogram of the PMMA homopolymer.

### 3.3. Characterization of Blends

To determine the effect of surface modification of cork particles, filaments of PMMA, Cork/PMMA and C-*g*-PMMA/PMMA blends were prepared. The morphology of the ensuing materials was studied by SEM, the mechanical properties by stress-strain tests, and the thermal properties by DMA and TGA. To assess the processability of the composites the MFI also was determined.

**Figure 5:**
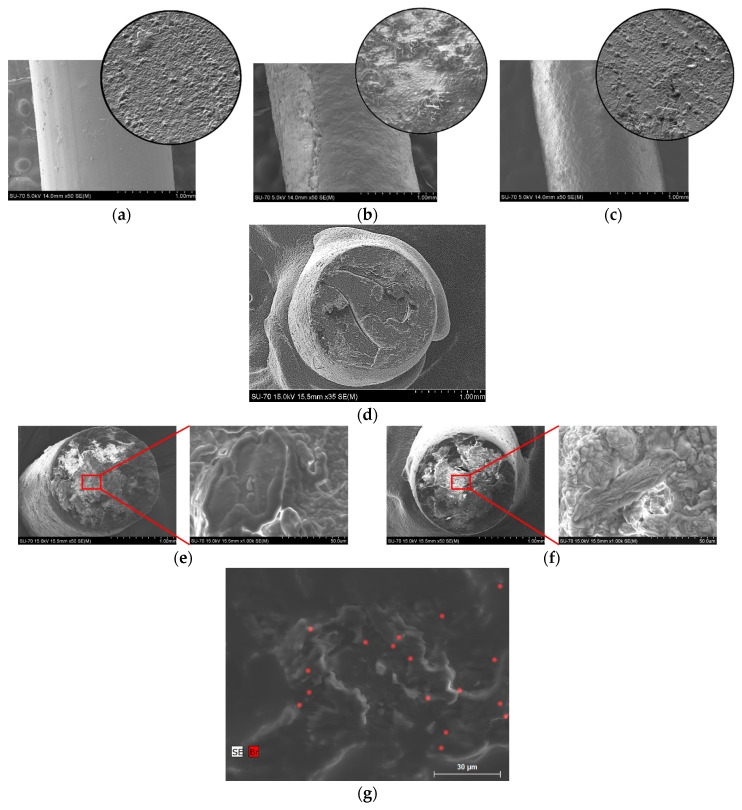
SEM images of the filaments: PMMA (**a**); Cork/PMMA (**b**), C-*g*-PMMA/PMMA (**c**), cross-section of the failure zone of filaments: PMMA (**d**); Cork/PMMA (**e**), C-*g*-PMMA/PMMA (**f**) as well as the EDX mapping of the failure zone of C-*g*-PMMA/PMMA (**g**).

Figure 5a–c shows the SEM images of the surface of the PMMA, Cork/PMMA and C-*g*-PMMA/PMMA filaments (with their cross-section highlighted), respectively. Moreover, in Figure 5d–f, the cross-section of the failure zone of the filaments after the mechanical tests is presented. Finally, the EDX mapping of the failure zone of C-*g*-PMMA/PMMA also is presented.

The SEM micrographs reveal that the surface of the filaments of the sample containing cork is rougher, indicating that the cork powder may not have been fully impregnated by PMMA. Actually, according to Stepashkin et al. [40], this limited impregnation might be due to the short residence time of the blend (melted PMMA and the filler) in the extruder. Yet, no voids are observed, which suggests good wettability of the cork by PMMA. Furthermore, the absence of voids which are normally associated with the start of material failure under stress is a promising feature regarding the mechanical properties of these materials. Considering the cross-section images of the filaments, the presence of the cork particles can be observed, especially in the case of Cork/PMMA, suggesting that the modification of cork results in a better distribution of cork in the filaments. Particularly, the bromine mapping of the failure zone of C-*g*-PMMA/PMMA, shown in Figure 5g, clearly proves that the cork particles modified by ATRP are well dispersed.

An important issue concerning the use of composites and blends in 3D printed technology is their processability, which can be estimated by measuring its MFI [41]. Seen in Table 3, the addition of unmodified cork reduces the MFI of the material from 6.7 to 5.3 g·10 min^−1^ (a reduction around 20%). Normally, the addition of a filler to polymeric matrices limits their free mobility, increasing the material’s apparent viscosity. Actually, the effect of fillers on the reduction of MFI has been reported by several authors [42,43,44]. However, the addition of C-*g*-PMMA content only caused a slight reduction in the MFI to 6.2 g·10 min^−1^ (a reduction around 7%), which can be associated with the better compatibility between the filler and the matrix.

**Figure 6:**
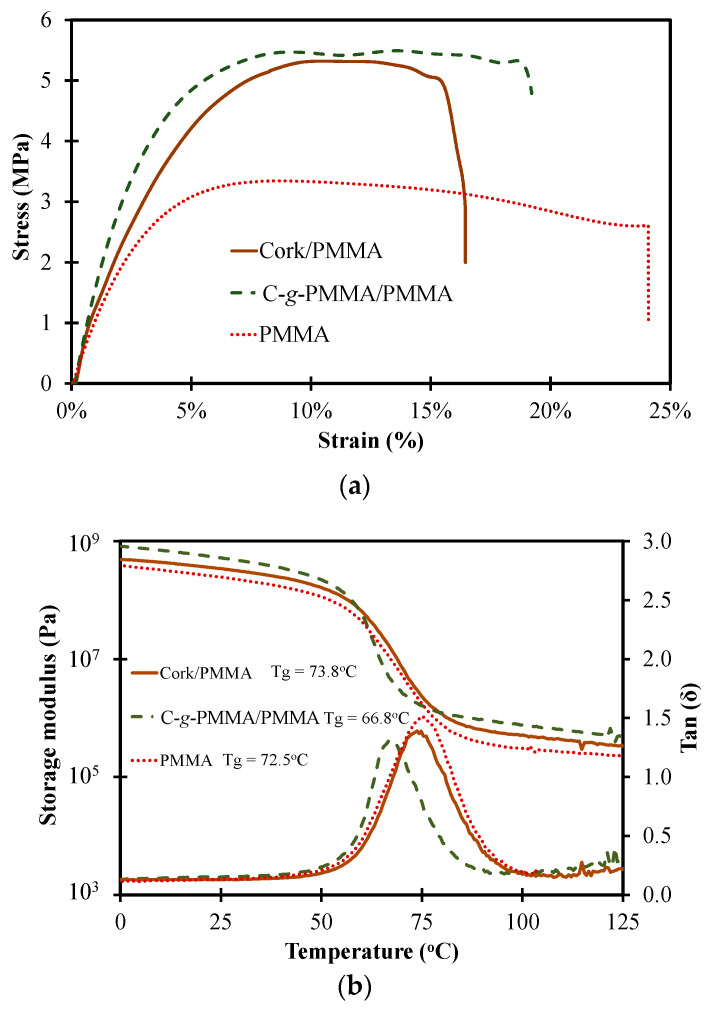
Static (**a**) and dynamic (**b**) mechanical properties of PMMA, Cork/PMMA and C-*g*-PMMA/PMMA filaments.

Regarding the mechanical properties, the static and dynamic mechanical properties of the filaments were measured, and the results are presented in Figure 6 and Table 3.

Considering the results presented in Table 3 and Figure 6a, it can be seen that the neat PMMA filament presents a Young’s modulus of 127.6 ± 1.0 MPa and an elongation at break of 24.1% ± 0.6, whereas the filaments filled with cork and C-*g*-PMMA present a Young’s modulus of 130.3 ± 2.0 MPa and 142.4 ± 3.2 MPa and an elongation at break of 17.1% ± 0.5 and 18.9% ± 0.4, respectively. It also can be observed that the neat filament presents a maximum stress of 3.3 ± 0.4 MPa and a toughness of 6151 ± 511 J·m^−3^, whereas the filaments filled with cork and C-*g*-PMMA present a maximum stress of 4.8 ± 1.1 MPa and 5.1 ± 0.6 MPa and a toughness of 5663 ± 971 J·m^−3^ and 6853 ± 228 J·m^−3^, respectively. The results obtained are consistent with the reinforcement of mixtures upon the addition of fillers, which generally results in stiffer materials and are in line with the results obtained by Fernandes et al. [22] who reported that the use of coupling agents in the preparation of cork–polymer composites improved the tensile strength of the ensuing materials, while cork increased the stiffness. Also, Abdallah et al. [21], who studied the effect of surface treatment of cork reinforced polypropylene (PP) composites, reported that the chemical treatment of the cork granules positively influences the cork/PP composite mechanical properties due to the better adhesion between the filler/matrix interface, increasing their tensile modulus. Furthermore, the higher Young’s modulus, elongation at break, maximum stress, and toughness of modified cork composites (when compared to the untreated cork-derived composites) can be associated with the better interface adhesion between matrix and filler. Indeed, a good interface promotes good transference of stress from the matrix to the filler, resulting in good mechanical performance. Actually, according to Wan et al. [24], the interphase zone may have contributed to increase the effective volume fraction of the filler.

DMA experiments also were carried out to obtain further information on the viscoelastic properties of the materials and the results are presented in Figure 6b. Occurring at lower temperatures (0 °C), the materials behave as hard solids, showing high storage modulus (E’) values: 3.9 × 10^8^, 4.9 × 10^8^, and 8.1 × 10^8^ kPa for PMMA, Cork/PMMA and C-*g*-PMMA/PMMA, respectively. The DMA results are in line with the static mechanical results: the stiffness of the materials increased as a result of the addition of the fillers and the highest storage modulus obtained is that of the specimen prepared using the modified cork sample. The increase of the storage modulus can be related to the filler-reinforcing effect of cork, as discussed previously. Above the glass transition temperature (*T_g_*) (measured at the top of the tan (δ)), the increase in molecular motion leads to the sharp reduction of the storage modulus to drop. The addition of fillers to matrices generally hinders chain mobility, and for that reason the *T_g_* increases. However, this is not always the case as other factors can have different effects on the glass transition temperature. While the addition of unmodified cork led to a slight increase of the *T_g_* value when compared to that of PMMA (1.3 °C), the modified cork caused a greater reduction of the *T_g_* (5.7 °C). This reduction is attributed to the grafted PMMA chains on the cork surface which, due to the affinity with the chains of the matrix, do not hinder their movements but, instead, seem to have a synergistic effect. Moreover, this result is in agreement with the MFI results obtained for this material.

**Figure 7:**
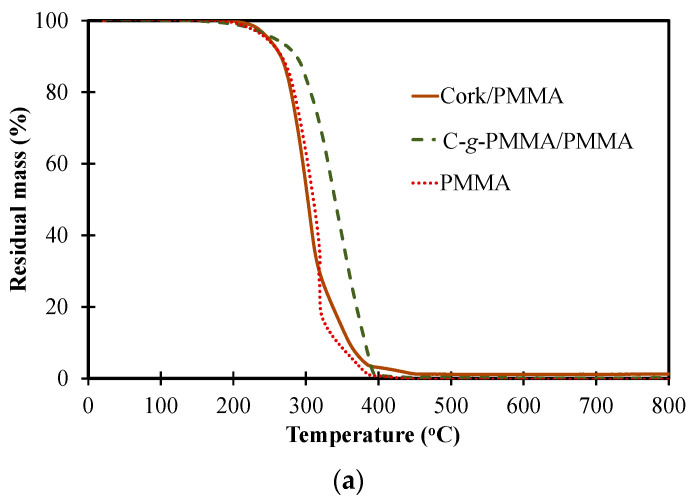

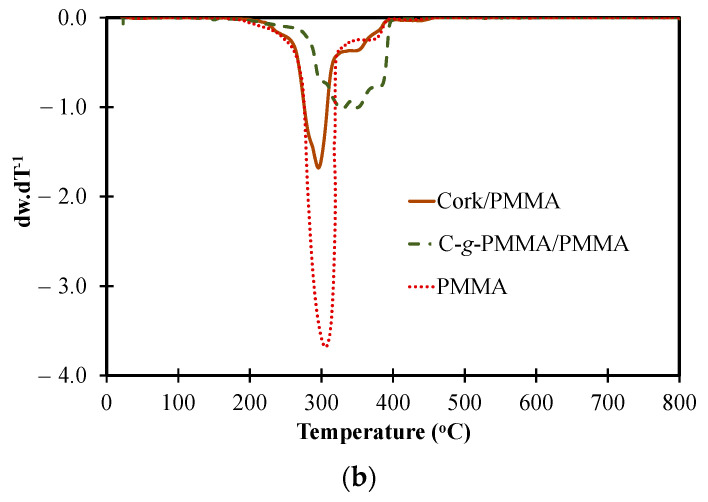
Thermal analyses: thermogram (**a**,**b**) first derivative of weight loss as a function of the temperature of Cork/PMMA, C-*g*-PMMA/PMMA and PMMA.

TGA also was carried out to evaluate the thermal stability of the materials and the thermograms obtained are presented in Figure 7.

Seen in Figure 7, the thermal decomposition of neat PMMA is characterized by a single degradation step, presenting a maximum of degradation around 300 °C, attributed to its decomposition to the methyl methacrylate (MMA) monomer [45]. Regarding the cork-containing materials, it is clear that C-*g*-PMMA/PMMA has a distinct degradation pattern. Actually, the 10% of mass loss is achieved at 265 °C for PMMA and Cork/PMMA, while the same amount of mass loss is only achieved at 287 °C for the C-*g*-PMMA/PMMA, indicating that the thermal stability of the material is enhanced using modified cork. Kiziltas et al. [46] produced PMMA/cellulose composites and reported initial degradation at higher temperatures using this filler. This enhancement of the thermal degradation was attributed to hydrogen bonding between the hydroxyl groups of cellulose with the carbonyl groups of PMMA. However, in the present study case, no significant difference was observed between the degradation temperatures of PMMA and Cork/PMMA, hence the enhanced thermal degradation of C-*g*-PMMA/PMMA must be attributed to the grafted PMMA chains on the cork surface. This may be a result of the fact that, due to the better dispersion of the fillers resulting from the grafting of PMMA chains, the surface area of cork is higher, thus, is more available to stabilize radicals resulting from thermal degradation. Actually, a similar result was obtained by Sailaja [47] while studying the effect of kraft pulp grafted with PMMA on the thermal properties of kraft wood pulp/low-density polyethylene (LDPE) composites. According to this author, the grafted wood pulp was thermally more stable than ungrafted wood pulp at the initial stages.

**Figure 8:**
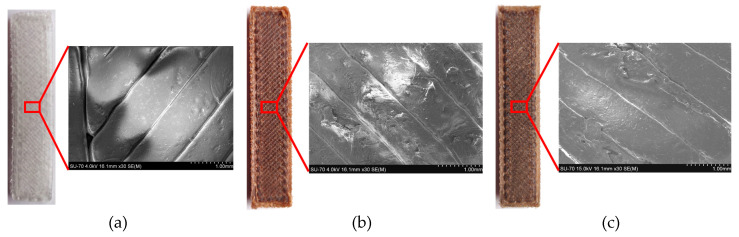
Photographs and SEM images of specimens: PMMA (**a**), Cork/PMMA (**b**) and C-*g*-PMMA/PMMA (**c**).

Finally, a preliminary test of the printability of these blends as 3D printing materials was carried out, and the 3D printed specimens and corresponding surface SEM images are presented in Figure 8.

Seen in Figure 8, the addition of cork to PMMA does not have any significant effect on the appearance of the 3D printed products, besides their color. Furthermore, in the SEM images of the 3D printed species voids were not observed. However, the surface of the specimen prepared using C-*g*-PMMA/PMMA is smoother than that of Cork/PMMA. Even though further studies will be needed to assess the applicability of the blends of PMMA and cork in 3D printed technology, the results obtained highlight the fact that using ATRP to graft PMMA chains from the surface of cork or other fillers can be a good strategy to improve the quality of 3D printed products.

Nevertheless, further systematic studies to optimize this strategy should include the determination of the amount of Br in the macroinitiator and its correlation with the average molecular weight of the grafted chains, as well as the preparation of macroinitiator samples with different levels of esterification, as well as the use of varying amounts of MMA to fully assess the best conditions and their impact on the resulting 3D products. Variations of ATRP to minimize the amount of copper catalyst in the final material also need to be investigated, as well as the effect of the terminal Br group on the graft chains to evaluate the influence of Br in the final material, in the blend, and dispersion in the PMMA matrix and, subsequently, in the 3D printing, thermal, and mechanics behavior.

## 4. Conclusions

Poly(methyl methacrylate) (PMMA) chains have been successfully grafted from cork particles by in situ atom transfer radical polymerization (ATRP). This strategy allowed a better dispersion of cork particles in PMMA-based blends as a result of the improved interface. The melt flow index (MFI), the mechanical and thermal properties of the materials prepared using PMMA-modified cork particles, were better than those obtained using bare cork particles. The modification of cork increased the MFI of the ensuing blends by 17%, the Young’s modulus by 10%, the maximum stress by 6%, and the toughness by 34%. Moreover, this surface modification also led to an increase of 22 °C at 10% of mass loss, when compared with that obtained using untreated cork particles. Although further systematic studies regarding the various factors discussed above are still required, it was demonstrated that it is possible to 3D print products from cork mixed with PMMA. Furthermore, it was shown that surface modification of cork particles yields smoother surfaces and an enhanced performance of the resulting materials. This proves that grafting using ATRP is a promising route to prepare high-quality 3D printed products.

## Figures and Tables

**Table 1:** 3D print parameters.

Parameter	Value
Layer Height	0.2 mm
Wall Thickness	1.2 mm
Infill Density	100%
Printing Temperature	175 °C
Build Plate Temperature	60 °C
Printing Speed	60 m·s^−1^

**Table 2:** TGA features of cork, C-B*i*B, C-*g*-PMMA and PMMA homopolymer.

Material	*T*_10%_ (°C) ^a^	*T*_dmax_ (°C) ^b^	*Wt*_800_ (%) ^c^
Cork	291	280–315, 329, 358, 412	16.9
C-B*i*B	243	230, 272, 333, 413	21.0
C-*g*-PMMA	287	381	6.3
PMMA ^d^	296	280, 291, 391	0.6

^a^ Temperature at 10% mass loss detected using TGA. ^b^ Temperature at maximum mass loss rate detected using DTG. ^c^ Weight of the solid residue remaining at 800 °C. ^d^ PMMA homopolymer (produced via ATRP).

**Table 3:** Mechanical properties of PMMA, Cork/PMMA and C-*g*-PMMA/PMMA filaments.

Sample	PMMA			Cork/PMMA			C-*g*-PMMA/PMMA		
MFI (g·10 min^−1^)	6.7	±	0.5	5.3	±	0.6	6.2	±	0.3
Young’s Modulus (MPa)	127.6	±	1.0	130.3	±	2.0	142.4	±	3.2
Elongation at break (%)	24.1	±	0.6	17.1	±	0.5	18.9	±	0.4
Maximum stress (MPa)	3.3	±	0.4	4.8	±	1.1	5.1	±	0.6
Toughness (J·m^−3^)	6151	±	511	5663	±	971	7616	±	1095
